# An Integrative Analysis Reveals the Underlying Association Between CTNNB1 Mutation and Immunotherapy in Hepatocellular Carcinoma

**DOI:** 10.3389/fonc.2020.00853

**Published:** 2020-06-12

**Authors:** Zhuomao Mo, Yongdan Wang, Zhirui Cao, Pan Li, Shijun Zhang

**Affiliations:** Department of Traditional Chinese Medicine, The First Affiliated Hospital, Sun Yat-sen University, Guangzhou, China

**Keywords:** hepatocellular carcinoma, CTNNB1, gene mutation, immunotherapy, immune inhibitor

## Abstract

**Background:** Tumor mutational burden (TMB) was verified to be closely associated with immune checkpoint inhibitors, but it is unclear whether gene mutation has an effect on immunotherapy of hepatocellular carcinoma (HCC). This research aimed to investigate the underlying correlation between gene mutation and immunotherapy in HCC.

**Methods:** The somatic gene mutation data and gene expression data were retrieved from International Cancer Genome Consortium database and The Cancer Genome Atlas (TCGA) database. The mutational genes were selected by the intersection of three cohorts and further identified using survival analysis and TMB correlation analysis. After the identification of key mutational gene, we explored the correlation between gene mutation and both the immune cell infiltration and immune inhibitors. The signaling pathways associated with gene mutation were confirmed through gene set enrichment analysis. Furthermore, the survival analysis and mutational analysis based on TCGA cohort were performed for the validation of included gene.

**Results:** As one of the frequently mutational genes in HCC, *CTNNB1* was finally included in our research, for which it showed the significant result in survival analysis and the positive association with TMB of the three cohorts. Meanwhile, the validation of TCGA showed the significant results. Furthermore, natural killer (NK) cells and neutrophil were found to significantly infiltrate *CTNNB1* mutation group from two cohorts. Besides, further analysis demonstrated that four types of immune inhibitors (*CD96, HAVCR2, LGALS9*, and *TGFB1*) were downregulated in *CTNNB1* mutation group.

**Conclusion:** Our research firstly revealed the underlying association between *CTNNB1* mutation and immunotherapy, and we speculated that *CTNNB1* mutation may modulate NK cells by affecting CD96. However, more functional experiments should be performed for verification.

## Introduction

Globally, liver cancer is a highly malignant tumor with high prevalence and poor outcomes, which results in ~850,000 new cases per year (1). As the major subtype of liver cancer, hepatocellular carcinoma (HCC) accounts for 85–90% of all liver cancer cases (1) and has become the second leading cause of cancer-associated deaths (2). It has been reported that the 5-year survival rate is 30.5% in patients with local HCC while <5% in patients with distant metastasis (3). At present, partial hepatectomy and liver transplantation are still the main treatments for early-stage patients, but a significant proportion of patients are not eligible for these treatments. Furthermore, the postoperative recurrence or distant metastasis is prevalent in patients after surgery (4). Although the systemic therapy with sorafenib is regarded as a first-line chemotherapeutic therapy in patients with advanced HCC, the high resistance rate has significantly limited the benefit of sorafenib therapy (5). Therefore, there is an urgent need to find a novel and effective therapy to improve the clinical outcomes of HCC patients.

The initiation, development, metastasis, and recurrence of HCC are closely related to the immune system (6). It has been reported that dysregulation of the immune system including alteration in the number or function of immune cells (7) and the release of chemokine and cytokine (8) result in the progression of HCC. Accordingly, immunotherapy has attracted increasing attention in HCC. As an important breakthrough in the field of immunotherapy, immune checkpoint inhibitors target three main molecules: cytotoxic T-lymphocyte-associated molecule-4 (*CTLA-4*), programmed cell death receptor-1 (*PD-1*), and programmed cell death ligand-1 (*PD-L1*) (9). It has been reported that camrelizumab showed antitumor activity and manageable toxicities in advanced HCC patients by blocking the interaction between *PD-1* and its ligands (10). Another clinical trial (11) also indicated that tremelimumab (*CTLA-4* blockade) showed antitumor and antiviral activities in advanced HCC patients. Nevertheless, only a minority of patients can respond to these immunotherapies, and fewer still achieve a lasting response (12). Consequently, it is one of the critical challenges to explore the molecular mechanism of immunotherapeutic responsiveness in HCC.

Tumor mutational burden (TMB) was defined as the total number of errors in somatic gene coding, base substitution, gene insertion, or deletion detected in every million base. Accumulation of somatic mutation contributes to the occurrence of tumor and the expression of neoantigens (13). Meanwhile, it has been reported that TMB can be used to predict the efficacy of immune checkpoint blockade and become a useful biomarker in some cancers for identification of patients who will benefit from immunotherapy (14). However, the potential association between gene mutation and immunotherapy in HCC is still unclear.

In this research, we firstly identified that *CTNNB1* was one of the frequently mutated genes in HCC and highly associated with survival and TMB. Next, we explored the relationship between *CTNNB1* mutation and immune cell infiltration and found that natural killer (NK) cells significantly infiltrated the *CTNNB1* mutation group. Therefore, we further investigated the correlation between *CTNNB1* mutation and immune inhibitors. We finally found that *CD96* was negatively associated with CTNNB1 mutation and speculated that *CTNNB1* may modulate NK cells by affecting *CD96*. Our research proposed a new underlying association between *CTNNB1* mutation and immunotherapy in HCC, which may help in improving the efficacy of immunotherapy in HCC patients.

## Materials and Methods

### Data Collection

The somatic gene mutation data, gene expression data, and clinical messages were retrieved from International Cancer Genome Consortium (ICGC) database (https://dcc.icgc.org/) and The Cancer Genome Atlas (TCGA) database (https://portal.gdc.cancer.gov/). Three independent cohorts (LIRI-JP, LICA-FR, and LINC-JP) in ICGC database were employed in our research. All the three cohorts were used for mutational gene selection, and the LIRI-JP cohort was employed for further analyses (owing to gene expression data and more known clinical parameters). In addition, the TCGA-LIHC cohort was used for further validation.

### Selection of Key Mutational Genes

Based on the “GenVisR” package under the R studio software, the details of mutation from the three cohorts were visualized in waterfall plot. After that, we employed the intersection of the three cohorts for further analyses and used the Venn plot to visualize. To investigate the time-dependent prognostic value of included genes, the survival analysis was performed using the “survival” package. Moreover, we explored the association between included genes and TMB. To calculate the TMB of each case, the total number of mutations counted was divided by the exome size (38 Mb was utilized as the exome size). The mutational genes were eligible for further analyses if they were significantly different in both the survival analysis and TMB correlation analysis. A *P*-value < 0.05 was considered a significant difference in this section. Besides, the mutational analysis and TMB correlation analysis were performed again based on the TCGA-LIHC cohort for validation.

### Immune Cell Infiltration and Immune Inhibitors

To explore the underlying mechanism between mutational gene and immune cells, we estimated the abundance of immune cell infiltration with different mutational status in the cases of the LIRI-JP cohort on the basis of the CIBERSORT algorithm. CIBERSORT is a deconvolution algorithm that evaluates the proportions of 22 tumor-infiltrating lymphocyte subsets. The number of permutations was set to 1,000, and the sample in the cohort was eligible for further validation if a *P*-value < 0.05. Meanwhile, the results of immune cell infiltration were verified in TIMER website (http://timer.cistrome.org/) on the basis of the TCGA-LIHC cohort. Besides, we investigated the correlation between 22 types of immune cells and survival. In addition, we evaluated the correlation between gene mutation and the expression of immunoinhibitory genes from TISIDB website (http://cis.hku.hk/TISIDB/index.php) (15). Both *P* < 0.05 and mean difference of median-value > 0.6 were considered significant association in TISIDB website.

### Gene Set Enrichment Analysis

Gene set enrichment analysis (GSEA) is a computational method that identifies whether a prior defined set of genes shows statistically significant differences between two biological states (16). In this research, we performed the GSEA to identify statistically different pathways from Gene Ontology (GO) and Kyoto Encyclopedia of Genes and Genomes (KEGG) databases between the mutation group and wild-type group. The normalized enrichment score was used to evaluate the pathways, and the top 5 significant pathways in two groups were visualized using the “ggplot” package.

## Results

### Identification of Key Mutational Genes

**Figure 1:**
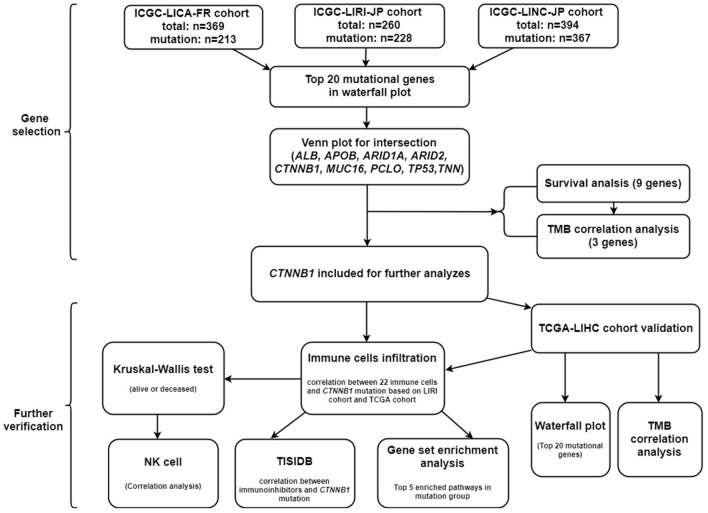
The flowchart of this study.

**Figure 2:**
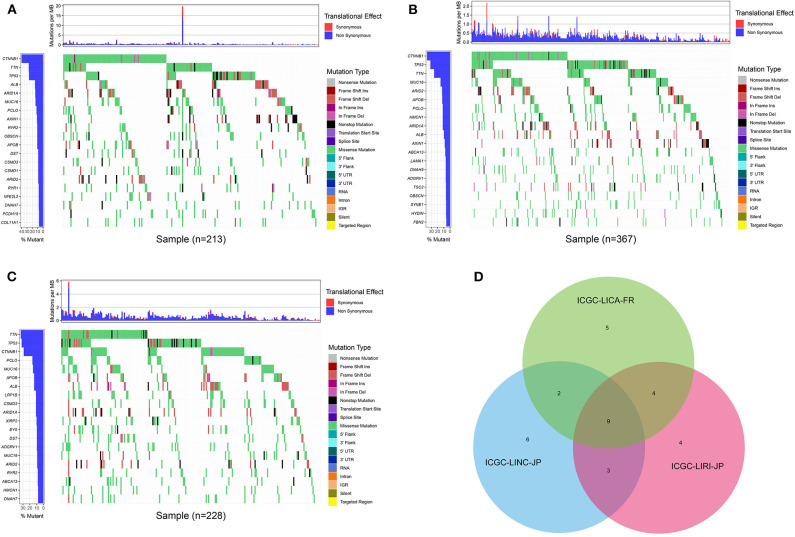
Waterfall plot with the details of gene mutation from three cohorts. **(A)** The waterfall plot of LICA-FR. **(B)** The waterfall plot of LINC-JP. **(C)** The waterfall plot of LIRI-JP. **(D)** The Venn plot of three cohorts.

**Figure 3:**
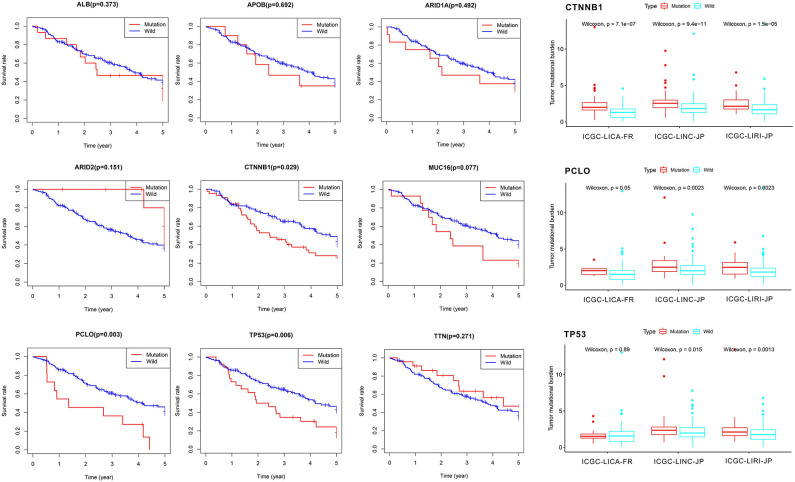
Survival analysis and tumor mutational burden (TMB) correlation analysis.

**Figure 4:**
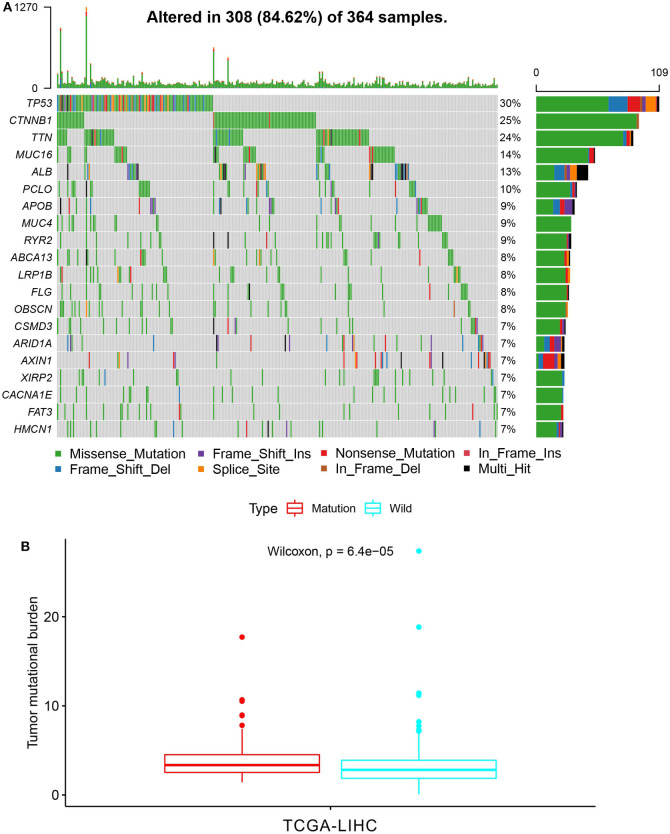
Validation based on TCGA-LIHC cohort. **(A)** The waterfall plot of TCGA-LIHC. **(B)** Tumor mutational burden (TMB) correlation analysis.

First of all, we summarized the flowchart, as shown in Figure 1. The clinical details of the ICGC cohorts are shown in Table 1. As illustrated in Figure 2, the details of the top 20 most frequently mutated genes are demonstrated in waterfall plots. Interestingly, we observed that some genes frequently mutated in all the three cohorts. So we executed a comparative analysis of the 20 most frequently mutated genes among the three cohorts. The Venn plot in Figure 2D shows that nine genes (*ALB, APOB, ARID1A, ARID2, CTNNB1, MUC16, PCLO, TP53*, and *TTN*) were included in the intersection of the three cohorts. After that, we performed a survival analysis to evaluate nine genes. The results of the survival analysis in Figure 3 indicated that a significant difference was found between the mutation group and wild-type group in the three genes (*CTNNB1, PCLO*, and *TP53*). Furthermore, we evaluated the correlation between the three genes and TMB, and the results indicated that only *CTNNB1* mutation was statistically significant with TMB in the three cohorts. Therefore, we focused on *CTNNB1* mutation in subsequent analyses. In addition, the validation of the TCGA-LIHC cohort demonstrated that *CTNNB1* was one of the frequently mutated genes (Figure 4A) and positively related to TMB in HCC (Figure 4B).

**Table 1:** Baseline patient characteristic in three cohorts.

**Clinical characteristics**		**Number**	**Percent**
**LICA-FR (*****n*** **=** **369)**			
Survival status	Survival	92	25
	Death	115	31
	Not reported	162	44
Age	≤65 years	205	56
	>65 years	164	44
Gender	Female	76	21
	Male	293	79
T classification	T1	54	14.6
	T2	65	17.6
	T3	40	10.8
	T4	1	0.3
	Not reported	209	56.7
N classification	N0	160	43
	N1	0	0
	Not reported	209	57
M classification	M0	159	43
	M1	1	0.3
	Not reported	209	56.7
**LINC-JP (*****n*** **=** **394)**			
Survival status	Survival	269	68
	Death	79	20
	Not reported	46	12
Age	≤65 years	175	45
	>65 years	206	52
	Not reported	13	3
Gender	Female	95	24
	Male	299	76
Stage	I	16	4.1
	II	69	17.5
	III	67	17
	IV	43	10.9
	Not reported	199	50.5
**LIRI-JP (*****n*** **=** **260)**			
Survival status	Survival	214	82.4
	Death	46	17.6
Age	≤65 years	98	37.7
	>65 years	162	62.3
Stage	I	40	15.4
	II	117	45
	III	80	30.8
	IV	23	8.8

### Immune Cell Infiltration and Immune Inhibitors

**Figure 5:**
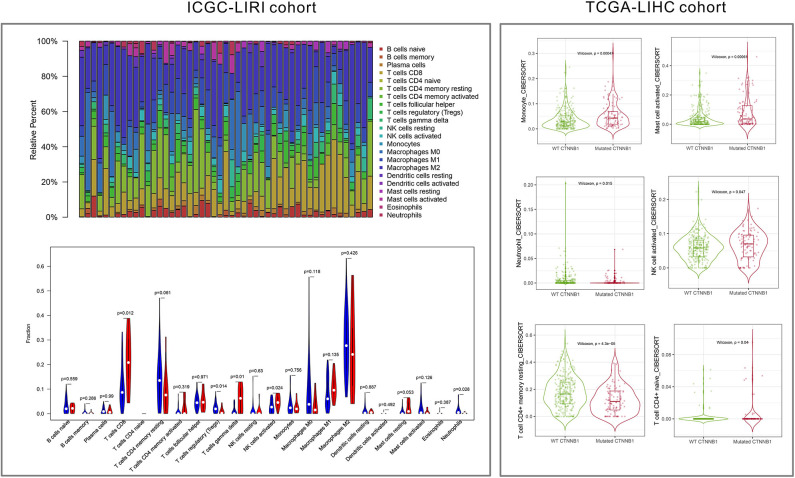
Immune cell infiltration from LIRI cohort and LIHC cohort.

**Figure 6:**
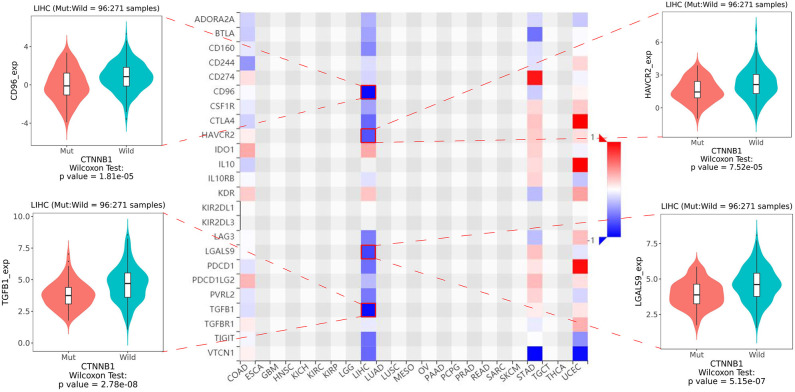
The heatmap of 24 types of immune inhibitors and significant results from correlation analysis.

**Figure 7:**
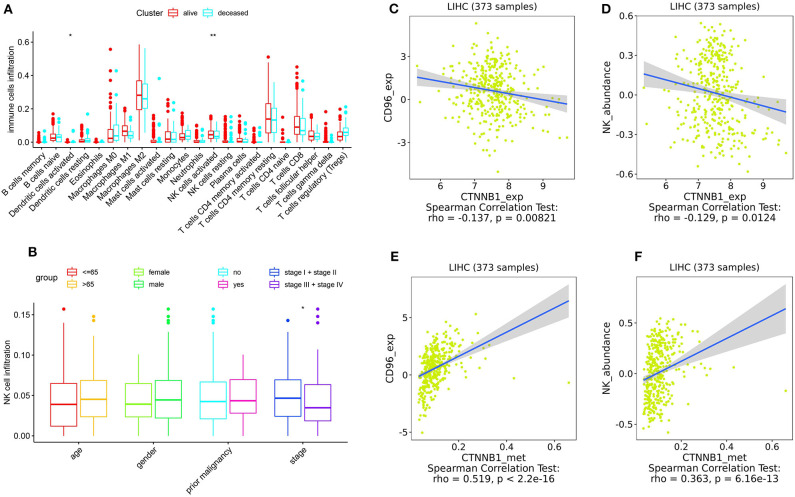
Correlation analysis. **(A)** The correlation between 22 types of immune cells and survival. **(B)** The correlation between NK cells and clinical parameters. **(C,D)** The correlation between CTNNB1 expression and CD96, and NK cells, respectively. **(E,F)** The correlation between CTNNB1 methylation and CD96, and NK cells, respectively. Notes: “*” in the plots means *P* < 0.05, “**” means *P* < 0.01.

As shown in Figure 5, the relative percent of 22 immune cell infiltration was visualized based on the LIRI-JP cohort. Between the mutation group and wild-type group, significant differences were found (*P* < 0.05) in five types of immune cells (CD8 T cells, regulatory T cells, gamma delta T cells, activated NK cells, and neutrophils) on the basis of the LIRI-JP cohort (Figure 5). In the TCGA-LIHC cohort, significant differences were found (Figure 5) in six types of immune cells (mast cell activated, monocyte, neutrophil, NK cell activated, T cell CD4^+^ memory resting, and T cell CD4^+^ naive). Both NK cell activated and neutrophil were significantly infiltrated the mutation group of two cohorts. In addition, the results of Figure 6 show that CTNNB1 mutation was negatively associated with *CD96, HAVCR2, LGALS9*, and *TGFB1*. And the expression difference of median between the two groups was −0.965 (*CD96*), −0.679 (*HAVCR2*), −0.733 (*LGALS9*), and −0.951 (*TGFB1*). Moreover, Figure 7A demonstrates that a significant difference was found between the live group and deceased group in dendritic cell activated and NK cell activated. Consequently, we focused on NK cells and further explored the correlation between NK cells and clinical parameters. The results from Figure 7B show that a significantly positive correlation was found between stage and NK cell infiltration in HCC. The verification from Figures 7C–F also indicates that the expression and methylation of CTNNB1 were significantly associated with CD96 expression and NK cell abundance.

### Underlying Pathways Associated With CTNNB1 Mutation

**Figure 8:**
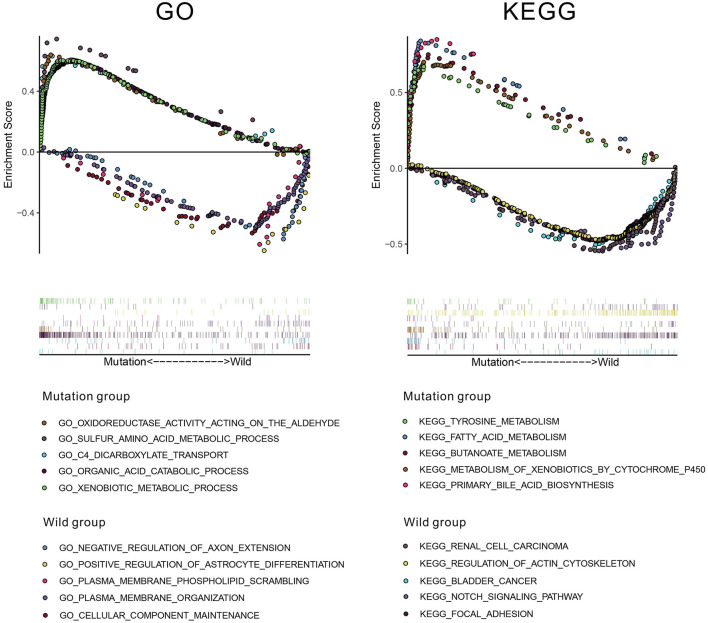
The results of gene set enrichment analysis (GSEA) based on Gene Ontology (GO) and Kyoto Encyclopedia of Genes and Genomes (KEGG) databases.

To investigate underlying pathways of GO and KEGG, we used GSEA to find significantly enriched terms by comparing the mutation and wild-type groups. We selected the 10 most relevant pathways according to the normalized enrichment score (five pathways in the mutation group and five in the wild-type group). As illustrated in Figure 8, 10 relevant pathways (oxidoreductase activity acting on the aldehyde or oxo group of donors, sulfur amino acid metabolic process, C4 dicarboxylate transport, organic acid catabolic process, and xenobiotic metabolic process in GO; tyrosine metabolism, fatty acid metabolism, butanoate metabolism, metabolism of xenobiotics by cytochrome P450, and primary bile acid biosynthesis in KEGG) are enriched in the mutation group, whereas other 10 pathways (negative regulation of axon extension, positive regulation of astrocyte differentiation, plasma membrane phospholipid scrambling, plasma membrane organization, and cellular component maintenance in GO; renal cell carcinoma, regulation of actin cytoskeleton, bladder cancer, notch signaling pathway, and focal adhesion in KEGG) enriched in the wild-type group.

## Discussion

With the increasing exploration of the immune system, immunotherapy was considered to have a crucial role in treatment of cancer. It has been verified that TMB was closely related to immune checkpoint inhibitors. However, the mechanism between gene mutation and immunotherapy in HCC was still unclear. Consequently, in our research, we firstly analyzed the details of gene mutation from the three cohorts. Then we performed a comparative analysis to find the intersection of the three cohorts, and we found that only three mutational genes were significantly associated with overall survival. Among the three genes, only *CTNNB1* was significantly associated with TMB in all three cohorts. Therefore, *CTNNB1* was selected for further investigation. *CTNNB1* (catenin beta-1) is a key regulatory molecule of canonical Wnt signaling pathway. An activating mutation in exon 3 of *CTNNB1* results in accumulation of β-catenin in the nucleus and activates the transcription of downstream target gene such as lymphoid enhancer-binding factor 1 (*LEF1*) (17), and *LEF1* is a transcription factor that has been implicated in the pathogenesis of multiple tumors (18). It has been reported that *CTNNB1* mutation was highly associated with many kinds of human tumors, such as biliary tract cancer (19), lung adenocarcinoma (20), and endometrioid ovarian carcinoma (21). In our research, *CTNNB1* mutation was significantly associated with a better prognosis and a higher TMB. A higher TMB leads to the exposure of more neoantigens, which may cause a T cell-dependent immune response (22). Meanwhile, previous studies (23, 24) indicated that *CTNNB1* significantly mutated in immune subtypes of HCC. As a result, *CTNNB1* mutation may have an effect on immunotherapy. To further explore the mechanism between *CTNNB1* mutation and immunotherapy, we compared the difference of immune cell infiltration between the mutation group and wild-type group. Interestingly, we found that NK cells significantly positively infiltrated the mutation group. Meanwhile, the results indicated that more NK cells significantly infiltrated the survival group. Accordingly, we focused on NK cells in subsequent analyses.

NK cell is an important part of the innate immune system, which can secrete cytokines and cytolytic activity against target cells. It has been verified that NK cells can efficiently eradicate heterogeneous tumor cells after a long-term treatment (25). Concerning HCC, the lack of NK cell number and the defects of NK cell function facilitated the escape of tumor cells from immune surveillance (26). In patients with advanced-stage HCC, NK cells were significantly decreased in number with impaired tumor necrosis factor alpha (TNF-α) and interferon-gamma (IFN-γ) production (27). Our results also showed the positive correlation between NK cell infiltration and stage. However, no significant correlation was found between NK cell infiltration and other clinical parameters like age and gender, which may attribute to the small sample size. Based on the implication of CTNNB1 mutation and NK cells in HCC, we speculated that *CTNNB1* mutation may enhance the effect of immunotherapy by NK cells.

Furthermore, we investigated the correlation between *CTNNB1* mutation and immunoinhibitory genes. In our research, we found that *CTNNB1* mutation was negatively associated with four immunoinhibitory genes. Among them, *CD96* is the novel immune checkpoint receptor in NK cells (28). Accumulating data support the targeting of *CD96* for improving antitumor immune response (29). Galectin-9 (*LGALS9*) is the most relevant ligand that interacts with Tim-3 (*HAVCR2*) (30). Binding of Tim-3 (*HAVCR2*) to galectin-9 (*LGALS9*) leads to Th1 cell death by apoptosis (30). Meanwhile, Tim-3 (*HAVCR2*) is an inducible human NK cell receptor that enhances IFN-γ production in response to galectin-9 (*LGALS9*) (31). In terms of *TGFB1*, it has been reported that *TGFB1* suppresses the function of NK cells by inducing miRNA23a (32). Four types of immune inhibitors showed the close association with NK cells, which verified the relationship between *CTNNB1* mutation and NK cells. On the one hand, our result indicated that *CD96* was most negatively correlated with CTNNB1 mutation. On the other hand, not only the mutation but also the expression and methylation of *CTNNB1* significantly related to *CD96* and NK cells. Consequently, we speculated that there is an underlying interactive association among CTNNB1, CD96, and NK cells. Considering the signaling pathways associated with *CTNNB1* mutation, we employed GSEA to find the significantly enriched pathways in the mutation group. Although we did not find any pathways related to immune response, five pathways involved metabolism were observed. Immune activation is now understood to be fundamentally linked to intrinsic and/or extrinsic metabolic process. It has been reported that carbohydrate and amino acid metabolism are hallmarks for the innate immune cell activation and function (33). Meanwhile, immune cells exhibit various responses against different types of microbes, which seems to be associated with changes in energy metabolism (34). But it is uncertain whether *CTNNB1* mutation affects the immunotherapy through metabolic pathways.

To our knowledge, it is the first research that focused on the gene mutation and immunotherapy in HCC. Our research revealed the implication of *CTNNB1* mutation in the immunotherapy of HCC. Furthermore, because *CTNNB1* mutation positively associated with immune inhibitors, *CTNNB1* mutation may serve as the novel biomarker in identifying the patients who will benefit from immune checkpoint blockade treatment. Nevertheless, some limitations in our research have to be pointed out. First, NK cells can be divided into subsets based on the expression of CD56 and CD16. It is necessary to investigate the mechanism among different NK cell subsets, CTNNB1 and CD96 in HCC. Second, the results of GSEA were preliminary; the current evidences of pathways still need to be validated in clinical trials and functional experiments.

## Conclusion

Our research firstly revealed the underlying association between *CTNNB1* mutation and immunotherapy, and we speculated that *CTNNB1* mutation may modulate NK cells by affecting CD96. However, more functional experiments should be performed for verification.

## Data Availability Statement

Publicly available datasets were analyzed in this study. This data can be found here: TCGA database (https://portal.gdc.cancer.gov/) and ICGC database (https://dcc.icgc.org/).

## Author Contributions

ZM and SZ designed the manuscript. ZM and YW wrote and completed the manuscript. ZC and PL completed the data download and analysis. All the authors approved the final manuscript.

## Conflict of Interest

The authors declare that the research was conducted in the absence of any commercial or financial relationships that could be construed as a potential conflict of interest.

## References

[1] Llovet JM Zucman-Rossi J Pikarsky E Sangro B Schwartz M Sherman M. Hepatocellular carcinoma. Nat Rev Dis Primers. (2016) 2:16018. 10.1038/nrdp.2016.18 27158749

[2] Ferlay J Soerjomataram I Dikshit R Eser S Mathers C Rebelo M. Cancer incidence and mortality worldwide: sources, methods and major patterns in GLOBOCAN 2012. Int J Cancer. (2015) 136:E359–86. 10.1002/ijc.29210 25220842

[3] Oweira H Petrausch U Helbling D Schmidt J Mehrabi A Schöb O. Prognostic value of site-specific extra-hepatic disease in hepatocellular carcinoma: a SEER database analysis. Expert Rev Gastroenterol Hepatol. (2017) 11:695–701. 10.1080/17474124.2017.1294485 28276812

[4] Bruix J Gores GJ Mazzaferro V. Hepatocellular carcinoma: clinical frontiers and perspectives. Gut. (2014) 63:844–55. 10.1136/gutjnl-2013-306627 24531850 PMC4337888

[5] Niu L Liu L Yang S Ren J Lai PBS Chen GG. New insights into sorafenib resistance in hepatocellular carcinoma: responsible mechanisms and promising strategies. Biochim Biophys Acta Rev Cancer. (2017) 1868:564–70. 10.1016/j.bbcan.2017.10.002 29054475

[6] Shi Y Men X Li X Yang Z Wen H. Research progress and clinical prospect of immunocytotherapy for the treatment of hepatocellular carcinoma. Int Immunopharmacol. (2020) 82:106351. 10.1016/j.intimp.2020.106351 32143005

[7] Ma C Kesarwala AH Eggert T Medina-Echeverz J Kleiner DE Jin P. NAFLD causes selective CD4(+) T lymphocyte loss and promotes hepatocarcinogenesis. Nature. (2016) 531:253–7. 10.1038/nature16969 26934227 PMC4786464

[8] Ma HY Yamamoto G Xu J Liu X Karin D Kim JY. IL-17 signaling in steatotic hepatocytes and macrophages promotes hepatocellular carcinoma in alcohol-related liver disease. J Hepatol. (2019) 72:946–59. 10.1016/j.jhep.2019.12.016 31899206 PMC7167339

[9] Peeraphatdit TB Wang J Odenwald MA Hu S Hart J Charlton MR. Hepatotoxicity from immune checkpoint inhibitors: a systematic review and management recommendation. Hepatology. (2020). 10.1002/hep.31227. [Epub ahead of print]. 32167613

[10] Qin S Ren Z Meng Z Chen Z Chai X Xiong J. Camrelizumab in patients with previously treated advanced hepatocellular carcinoma: a multicentre, open-label, parallel-group, randomised, phase 2 trial. Lancet Oncol. (2020) 21:571–80. 10.1016/S1470-2045(20)30011-5 32112738

[11] Sangro B Gomez-Martin C De La Mata M Inarrairaegui M Garralda E Barrera P. A clinical trial of CTLA-4 blockade with tremelimumab in patients with hepatocellular carcinoma and chronic hepatitis C. J Hepatol. (2013) 59:81–8. 10.1016/j.jhep.2013.02.022 23466307

[12] Braun DA Burke KP Van Allen EM. Genomic approaches to understanding response and resistance to immunotherapy. Clin Cancer. (2016) 22:5642–50. 10.1158/1078-0432.CCR-16-0066 27698000 PMC5135569

[13] Gubin MM Artyomov MN Mardis ER Schreiber RD. Tumor neoantigens: building a framework for personalized cancer immunotherapy. J Clin Invest. (2015) 125:3413–21. 10.1172/JCI80008 26258412 PMC4588307

[14] Chan TA Yarchoan M Jaffee E Swanton C Quezada SA Stenzinger A. Development of tumor mutation burden as an immunotherapy biomarker: utility for the oncology clinic. Ann Oncol. (2019) 30:44–56. 10.1093/annonc/mdy495 30395155 PMC6336005

[15] Ru B Wong CN Tong Y Zhong JY Zhong SSW Wu WC. TISIDB: an integrated repository portal for tumor-immune system interactions. Bioinformatics. (2019) 35:4200–2. 10.1093/bioinformatics/btz210 30903160

[16] Subramanian A Tamayo P Mootha VK Mukherjee S Ebert BL Gillette MA. Gene set enrichment analysis: a knowledge-based approach for interpreting genome-wide expression profiles. Proc Natl Acad Sci USA. (2005) 102:15545–50. 10.1073/pnas.0506580102 16199517 PMC1239896

[17] Taketo MM. Shutting down Wnt signal-activated cancer. Nat Genet. (2004) 36:320–2. 10.1038/ng0404-320 15054482

[18] Suzuki Y Ichihara S Kawasaki T Yanai H Kitagawa S Shimoyama Y. β-catenin (CTNNB1) mutation and LEF1 expression in sinonasal glomangiopericytoma (sinonasal-type hemangiopericytoma). Virchows Arch. (2018) 473:235–9. 10.1007/s00428-018-2370-9 29736797

[19] Hogdall D Larsen OF Linnemann D Svenstrup Poulsen T Hogdall EV. Exome sequencing of 22 genes using tissue from patients with biliary tract cancer. APMIS. (2020) 128:3–9. 10.1111/apm.13003 31628675

[20] Zhou C Li W Shao J Zhao J Chen C Analysis of the clinicopathologic characteristics of lung adenocarcinoma with mutation. Front Genet. (2019) 10:1367 10.3389/fgene.2019.01367 32117418 PMC7026668

[21] Pierson WE Peters PN Chang MT Chen LM Quigley DA Ashworth A. An integrated molecular profile of endometrioid ovarian cancer. Gynecol Oncol. (2020) 157:55–61. 10.1016/j.ygyno.2020.02.011 32139151

[22] Mcgranahan N Furness AJS Rosenthal R Ramskov S Lyngaa R Saini SK. Clonal neoantigens elicit T cell immunoreactivity and sensitivity to immune checkpoint blockade. Science. (2016) 351:1463–9. 10.1126/science.aaf1490 26940869 PMC4984254

[23] Li W Wang H Ma Z Zhang J Ou-Yang W Qi Y. Multi-omics analysis of microenvironment characteristics and immune escape mechanisms of hepatocellular carcinoma. Front Oncol. (2019) 9:1019. 10.3389/fonc.2019.01019 31681571 PMC6803502

[24] Wei L Delin Z Kefei Y Hong W Jiwei H Yange Z. A classification based on tumor budding and immune score for patients with hepatocellular carcinoma. Oncoimmunology. (2020) 9:1672495. 10.1080/2162402X.2019.1672495 32002283 PMC6959452

[25] Dianat-Moghadam H Rokni M Marofi F Panahi Y Yousefi M. Natural killer cell-based immunotherapy: from transplantation toward targeting cancer stem cells. J Cell Physiol. (2018) 234:259–73. 10.1002/jcp.26878 30144312

[26] Liu P Chen L Zhang H. Natural killer cells in liver disease and hepatocellular carcinoma and the NK cell-based immunotherapy. J Immunol Res. (2018) 2018:1206737. 10.1155/2018/1206737 30255103 PMC6142725

[27] Wu Y Kuang DM Pan WD Wan YL Lao XM Li XF. Monocyte/marcrophage-elicited natural killer cell dysfunction in hepatocellular carcinoma is mediated by CD48/2B4 interactions. Hepatology. (2013) 57:1107–16. 10.1002/hep.26192 23225218

[28] Kim N Kim HS. Targeting checkpoint receptors and molecules for therapeutic modulation of natural killer cells. Front Immunol. (2018) 9:2041. 10.3389/fimmu.2018.02041 30250471 PMC6139314

[29] Dougall WC Kurtulus S Smyth MJ Anderson AC. TIGIT and CD96: new checkpoint receptor targets for cancer immunotherapy. Immunol Rev. (2017) 276:112–20. 10.1111/imr.12518 28258695

[30] Zhu C Anderson AC Schubart A Xiong H Imitola J Khoury SJ. The Tim-3 ligand galectin-9 negatively regulates T helper type 1 immunity. Nat Immunol. (2005) 6:1245–52. 10.1038/ni1271 16286920

[31] Gleason MK Lenvik TR Mccullar V Felices M O'brien MS Cooley SA. Tim-3 is an inducible human natural killer cell receptor that enhances interferon gamma production in response to galectin-9. Blood. (2012) 119:3064–72. 10.1182/blood-2011-06-360321 22323453 PMC3321868

[32] Berchem G Noman MZ Bosseler M Paggetti J Baconnais S Le Cam E. Hypoxic tumor-derived microvesicles negatively regulate NK cell function by a mechanism involving TGF-β and miR23a transfer. Oncoimmunology. (2016) 5:e1062968. 10.1080/2162402X.2015.1062968 27141372 PMC4839360

[33] Zhao H Raines LN Huang SC-C. Carbohydrate and amino acid metabolism as hallmarks for innate immune cell activation and function. Cells. (2020) 9:E562. 10.3390/cells9030562 32121028 PMC7140477

[34] Hosomi K Kunisawa J. Diversity of energy metabolism in immune responses regulated by microorganisms and dietary nutrition. Int Immunol. (2020). 10.1093/intimm/dxaa020. [Epub ahead of print]. 32219308 PMC7318777

